# Facility assessment and qualitative analysis of health worker perspectives on neonatal health in Malawi

**DOI:** 10.1186/s13104-021-05679-5

**Published:** 2021-07-12

**Authors:** Mai-Lei Woo Kinshella, Alinane Linda Nyondo-Mipando, Queen Dube, David M. Goldfarb, Kondwani Kawaza

**Affiliations:** 1 Department of Obstetrics and Gynaecology, BC Children’s and Women’s Hospital and University of British Columbia, Vancouver, Canada; 2 Department of Health Systems and Policy, School of Public Health and Family Medicine, College of Medicine, University of Malawi, Blantyre, Malawi; 3 Pediatrics, Queen Elizabeth Central Hospital, Blantyre, Malawi; 4 Department of Pathology and Laboratory Medicine, BC Children’s and Women’s Hospital and University of British Columbia, Vancouver, Canada; 5 Department of Pediatrics and Child Health, College of Medicine, University of Malawi, Blantyre, Malawi

**Keywords:** Facility assessment, Qualitative research, Newborn care, Bubble continuous positive airway pressure (CPAP), Health worker perspectives, Malawi

## Abstract

**Objectives:**

The “Integrating a neonatal healthcare package for Malawi” (IMCHA#108030) project conducted mixed-methods to understand facility-based implementation factors for newborn health innovations in low-resourced health settings. The objective of the two datasets was to evaluate: (a) capacity of quality newborn care in three districts in southern Malawi, and (b) barriers and facilitators the scale up of bubble continuous positive airway pressure (CPAP), a newborn health innovation to support babies with respiratory distress.

**Data description:**

The Integrated Maternal, Neonatal and Child Quality of Care Assessment and Improvement Tool (version April-2014) is a standardized facility assessment tool developed by the World Health Organization (WHO) that examines quality as well as quantity and availability. The facility survey is complemented by a qualitative dataset of illustrative quotes from health service providers and supervisors on bubble CPAP implementation factors. Research was conducted in one primary health centre (facility assessment only), three district-level hospitals (both) and a tertiary hospital (qualitative only) in southern Malawi. These datasets may be used by other researchers for insights into health systems of low-income countries and implementation factors for the roll-out of neonatal health innovations as well as to frame future research questions or preliminary exploratory research on similar topics.

**Supplementary Information:**

The online version contains supplementary material available at 10.1186/s13104-021-05679-5.

## Objective

Neonatal mortality has become an increasingly important contributor to overall global child mortality and is now estimated to be over 45% of all under-5 mortalities [1]. In Africa alone, it is estimated that approximately 1 million babies per year die in their first 4 weeks of life [2]. Though Malawi, a small land-locked country in sub-Saharan Africa, is celebrated for achieving the Millennium Development Goals of reducing under-5 mortality by two-thirds, neonatal mortality rates remain high with latest estimates of 20 per 1000 live births, equating to 12,000 neonatal deaths in 2019 alone [3]. Malawi has an estimated preterm birth rate of 10.5% [4] and approximately 35% of neonatal deaths are attributed to preterm birth, making prematurity the leading direct cause of neonatal mortality [1]. Preterm infants pose particular challenges; they are especially vulnerable to feeding difficulties because their sucking and swallowing reflexes are immature, they develop breathing problems and have body temperature instability [5, 6].

In this project, we conducted mixed-methods research to understand how best to scale up essential life-saving neonatal interventions at the district-level in the Malawian context. The database includes a comprehensive facility survey for newborn care adapted from the World Health Organization (WHO) Integrated Maternal, Neonatal and Child Quality of Care Assessment and Improvement Tool (version-April-2014). The database also includes qualitative analyses of health worker perspectives on barriers and facilitators to implementing bubble continuous positive airway pressure (CPAP). Research was conducted in one primary health centre (facility assessment only), three district-level hospitals (both) and a tertiary hospital (qualitative only). The three district-level hospitals have coded numeric identifiers to link between the facility survey and qualitative datasets. An assessment of the quality of newborn care at district hospitals in Malawi was published from the facility survey dataset [7] and the qualitative dataset was used to understand perspectives of health workers on barriers and enablers of implementing bubble CPAP [8] and factors affecting caregiver engagement with bubble CPAP [9]. These datasets would be of value to researchers seeking to strengthen newborn care in other low resource settings. Connections between qualitative and quantitative datasets may lead to novel insights into factors that impact scaling up innovations.

## Data description

This research was collected as part of the “Integrating a neonatal healthcare package for Malawi” (IMCHA #108030), part of the Innovating for Maternal and Child Health in Africa (IMCHA) initiative.

Using the WHO Integrated Maternal, Neonatal and Child Quality of Care Assessment and Improvement Tool (version April-2014), the facility surveys were conducted health facilities providing secondary-level care in three districts in southern Malawi in November 2017. There were government hospitals in two districts and a private not-for-profit mission hospital alongside a public primary health centre that provided some secondary level services in the third district. The assessment tool was not modified for the primary health centre, though some inpatient services were skipped when it was not provided. Assessments in all facilities were conducted by trained data collectors, which involved observations of practices and resource availability as well as interviewing relevant health professionals, such as the nurse-in-charge of the ward and laboratory technicians (Additional file 1).

This study focuses only on neonatal care and includes the modules on infrastructure, neonatal care and maternal care related to labour and delivery. Using a series of structured checklists, each aspect of care was observed and scored 1–5. A score of five indicated good practice complying with WHO standards of care, a score of four indicated minor need for improvement, a score of three indicated some need for improvement, a score of two indicated considerable need and a score of one indicated totally inadequate care or a potentially life-threatening practice. Variables assessed included infrastructure, ward layout, organisation of care including staffing, emergency care, in patient care, infection control and supportive care, essential drugs, equipment and supplies, case management, and monitoring and follow up. Dimensions of the dataset are by facility described by type and linked with the qualitative dataset by coded identifier (District 1-government hospital, District 2-mission hospital, District 3-government hospital). With over 600 questions, the resulting data are a comprehensive evaluation of the capacity for neonatal care available in 2016 at secondary-level care facilities in rural southern Malawi. The resulting data are largely quantitative, with some open-text comments to qualify numbered responses. This survey used modules that dealt specifically with neonates from the WHO Integrated Maternal, Neonatal and Child Quality of Care Assessment and Improvement Tool and can be compared with other studies using the same tool.

The semi-structured interviews were conducted in June to August 2018. Face-to-face interviews were conducted by trained Malawian researchers at health facilities with 46 health workers. These included nurses, clinical officers, district health management (district health officer, district medical officer and district nursing officer), pediatric consultants and registrars. The qualitative dataset of health worker perspectives on barriers and facilitators to implementation of bubble CPAP includes the following variables: training and staffing, initiation, monitoring, weaning, caregivers and supplies and equipment. Dimensions of the dataset are by facility type and health worker cadre. Quotes include detail on the sex of the respondent and age (F for female, M for male and number for age). Health facility numbering (District 1-government hospital, District 2-mission hospital, District 3-government hospital) will be the same as in the facility survey to provide a link between the two datasets in this database. In addition, a tertiary-level hospital (Central hospital) is also included in the qualitative dataset. This data could be used by other researchers to understand barriers and facilitators to implementing innovative technologies in resource constrained settings and as a baseline for the scale up of neonatal care packages in Malawi (Table 1).

**Table 1:** Overview of data files/data sets

Label	Name of data file/data set	File types (file extension)	Data repository and identifier (DOI or accession number)
Data file 1	Detailed description of methodology	MS word file (.docx)	figshare (https://doi.org/10.6084/m9.figshare.12380480) [10]
Data set 1	Facility assessment.xlsx	MS Excel file (.xlsx)	figshare (https://doi.org/10.6084/m9.figshare.12380480) [10]
Data set 2	Qualitative dataset.csv	MS Excel file (.csv)	figshare (https://doi.org/10.6084/m9.figshare.12380480) [10]

## Limitations

To maintain participant’s privacy and prevent sharing identifying information, qualitative data are presented coded to general topic areas rather than raw transcripts. Quotes have been reviewed for identifying information and place names replaced by their facility type, participants are aggregated to their overall medical cadre category instead of specific positions and study sites are numbered instead of named. Consequently, qualitative responses are grouped as “nurses”, which includes nursing officer, senior nurses, registered nurses and nurse technicians, and “district health management” includes district health officer, district medical officer and district nursing officer to protect confidentiality.

## Supplementary Information

**Additional file 1: Table S1.** Structure of the full WHO Integrated Maternal, Neonatal and Child Quality of Care Assessment and Improvement Tool. **Table S2.** Areas of care assessed by the adapted WHO integrated quality of care assessment tool. **Table S3.** Study site characteristics.

## Data Availability

The data described in this Data note can be freely and openly accessed on the figshare repository https://doi.org/10.6084/m9.figshare.12380480. Please see Table 1 and reference [8] for details and links to the data.

## Abbreviations

CPAP: Continuous positive airway pressure
IMCHA: Innovating for Maternal and Child Health in Africa initiative
WHO: World Health Organization
