# Supplementary material for: Facility assessment and qualitative analysis of health worker perspectives on neonatal health in Malawi
Source: BMC Res Notes. 2021 Jul 12;14:267. doi: 10.1186/s13104-021-05679-5 (PMC8273934; doi:10.1186/s13104-021-05679-5)
Supplement: Supplementary file 1 — Additional file 1: Table S1. Structure of the full WHO Integrated Maternal, Neonatal and Child Quality of Care Assessment and Improvement Tool. Table S2. Areas of care assessed by the adapted WHO integrated quality of care assessment tool. Table S3. Study site characteristics. [file 13104_2021_5679_MOESM1_ESM.docx]

**Detailed description of methodology**

**Integrating a neonatal healthcare package for Malawi**

This research was collected as part of the “Integrating a neonatal healthcare package for Malawi” (IMCHA #108030), part of the Innovating for Maternal and Child Health in Africa (IMCHA) initiative funded by the Canadian International Development Research Centre (IDRC) in partnership with Global Affairs Canada (GAC) and the Canadian Institutes for Health Research (CIHR). With a focus on implementation science and quality improvement, the project seeks to strengthen neonatal care at health facilities in low-resource settings such as Malawian central and district hospitals through understanding the baseline capacity for quality newborn care and barriers and facilitators to implementing newborn innovations. These methods are expanded versions of descriptions in our related work (facility survey (1); qualitative dataset (2,3)).

**Facility survey**

***The WHO integrated quality of care assessment tool***

The WHO Integrated Maternal, Neonatal and Child Quality of Care Assessment and Improvement Tool was developed in 2014 from two existing survey instruments, the Health Facility Survey and the Hospital Care for Mothers and Newborn Babies quality assessment and improvement tool (4). Based on the Integrated Management of Childhood Illness (IMCI) clinical guidelines, the Health Facility Survey was developed by the WHO Department of Child and Adolescent Health and Development in 2003 to evaluate the quality of care delivered to sick children (5). First published in 2009 and revised in 2014 by the WHO Regional Office for Europe, the Hospital Care for Mothers and Newborn Babies quality assessment and improvement tool features a comprehensive systematic, standards-based, participatory approach (6). Integration of the two surveys generated a generic assessment tool to evaluate facility-based maternal, neonatal and pediatric quality of care. The WHO Integrated Maternal, Neonatal and Child Quality of Care Assessment and Improvement Tool was refined by prioritization of high impact areas to improve quality care and alignment with standards derived from the WHO Pocketbook of Hospital Care for Children and the WHO Integrated Management of Pregnancy and Childbirth (7,8). Triage, hygiene, emergency and first-line drug availability, availability of updated standard treatment guidelines and management of emergency, common and routine conditions was determined to be key priority areas of greatest impact (7,8). The WHO Integrated Maternal, Neonatal and Child Quality of Care Assessment and Improvement Tool is unique from other facility assessment tools in that it examines quality of care as well as quantity and availability and is intended for both assessment and quality improvement (4,7). The tool is meant to contribute to the identification of gaps in key areas of maternal, newborn and child health care that needs to be improved (7). See table 1 for the structure of the full tool.

***Table 1. Structure of the full WHO Integrated Maternal, Neonatal and Child Quality of Care Assessment and Improvement Tool***

| **Module** | **Topic area** | **Coverage** |
| --- | --- | --- |
| Module A | Infrastructure | - Lay out - Infrastructure - Staffing - Hospital statistics - Health information system and medical records - Essential drugs and blood products - Laboratory - Guidelines and auditing |
| Module B | Maternal care | - Emergency care - Wards - Infection control and supportive care - Essential drugs, equipment and supplies - Case Management - Monitoring and follow-up |
| Module C | Newborn care | - Emergency care - Wards - Infection control and supportive care - Essential drugs, equipment and supplies - Case Management - Monitoring and follow-up |
| Module D | Paediatric care | - Emergency care - Wards - Infection control and supportive care - Essential drugs, equipment and supplies - Case Management - Monitoring and follow-up |

We used the WHO Integrated Maternal, Neonatal and Child Quality of Care Assessment and Improvement Tool version April 2014, which was shared to us by project collaborators in the Ministry of Health, Government of Malawi. The tool (version April 2014) was previously used in Malawi (4), in Sierra Leone (9–12) and adopted as the assessment tool for hospital care by the WHO Regional Office of South-East Asia (8).

***Adaptation of the assessment tool***

The WHO Integrated Maternal, Neonatal and Child Quality of Care Assessment and Improvement Tool is developed as a generic framework to be adapted to the local context including epidemiology and health system structure factors (7,8). The structure of the tool sectioned into modules allows for its adaption of maternal, newborn of paediatric care as required by the country, hospital facility or project (7,8). Our study focused on neonatal care and facility assessments were a baseline for the overall project to understand newborn care environments and capacities at district-level facilities in Malawi. Consequently, only the modules on infrastructure, neonatal care and maternal care, as related to labour and delivery, were included. There were twelve key areas of care assessed covering infrastructure (A), laboratory (A), labour and delivery facilities (B), caesarean section facilities (B), prevention and management of preterm labour (B), nursery facilities (C), infection control (C), supportive care of sick neonates (C), neonatal care equipment and supplies (C), routine neonatal care (C), case management of the sick newborn (C) and monitoring and follow-up of sick newborns (C) (see table 2).

***Table 2. Areas of care assessed by the adapted WHO integrated quality of care assessment tool***

| **Area of care** | **Standards assessed** |
| --- | --- |
| Infrastructure | Availability of electricity, back-up power supply, running water, soap/disinfectant, sharps disposal, fridge for drugs, oxygen source, complaints/ suggestions box |
| Laboratory | Lab testing possible and results available for blood glucose, haemoglobin, hematocrit (PCV), HIV, syphilis, blood gas analysis, blood grouping and crossmatch, bilirubin, Rhesus antibody, urine dipstick, urine microscopy, blood culture, full blood count testing |
| Labour and delivery facilities | Presence of adequate lighting, Examination light, Wall clock, Delivery pack, Blank partographs, Heating lamp for neonates, Towels for drying newborn babies, Oxygen source: oxygen cylinder, Oxygen source: oxygen concentrator, Oxygen source: central supply, Flow meters for oxygen, Equipment for the administration of oxygen via nasal prongs, Equipment for the administration of oxygen via catheters, Equipment for the administration of oxygen via masks, Self-inflating bags for respiratory support, Neonatal size bags and masks, Anaesthetic equipment, Normal thermometer, Sterile gloves, Sterile gauze, Foetal stethoscope, Stethescope, Sphygmomanometer, Infusion sets, Infusion pumps/dosimeters, IV catheters, Urinary catheter, Syringes, Needles, Suturing set, Suturing material, Weighing scale for baby, Cord cutting/cord clamping set, Episiotomy scissors, Vacuum extractor, Forceps, Caesarean packs, Vacuum aspirator, Delivery beds, Regular beds, Operating theatre beds, Resuscitation table, Incubator, Tracheal tubes, Newborn bag and mask size 1 for term babies, Newborn bag and mask size 0 for pre-term babies, Laryngoscope blades, Oropharyngeal airways, Breathing valves, Electric suction pump, Suction catheter, Suction bulb, single use, Suction bulb, sterilizable multi-use, Baby scales, Hot cots, CPAP, Phototherapy |
| Caesarean section facilities | Presence of adequate lighting, Examination light, Wall clock, Delivery pack, Blank partographs, Heating lamp for neonates, Towels for drying newborn babies, Oxygen source: oxygen cylinder, Oxygen source: oxygen concentrator, Oxygen source: central supply, Flow meters for oxygen, Equipment for the administration of oxygen via nasal prongs, Equipment for the administration of oxygen via catheters, Equipment for the administration of oxygen via masks, Self-inflating bags for respiratory support, Neonatal size bags and masks, Anaesthetic equipment, Normal thermometer, Sterile gloves, Sterile gauze, Foetal stethoscope, Stethescope, Sphygmomanometer, Infusion sets, Infusion pumps/dosimeters, IV catheters, Urinary catheter, Syringes, Needles, Suturing set, Suturing material, Weighing scale for baby, Cord cutting/cord clamping set, Episiotomy scissors, Vacuum extractor, Forceps, Caesarean packs, Vacuum aspirator, Delivery beds, Regular beds, Operating theatre beds, Resuscitation table, Incubator, Tracheal tubes, Newborn bag and mask size 1 for term babies, Newborn bag and mask size 0 for pre-term babies, Laryngoscope blades, Oropharyngeal airways, Breathing valves, Electric suction pump, Suction catheter, Suction bulb, single use, Suction bulb, sterilizable multi-use, Baby scales, Hot cots, CPAP, Phototherapy |
| Prevention and management of preterm labour | Management of preterm labour and antenatal administration of corticosteriods |
| Nursery facilities | Availability of toilets, access to clean running water, clean and safe beds, mosquito nets, ward is clean, sharps container |
| Infection control | Hand hygiene, use of glovers, infection control practices |
| Supportive care of sick neonates | Supportive care including IV fluids use, drug treatment and blood transfusion |
| Neonatal care equipment and supplies | Availability of incubator, radiant warmer, a heated mattress cot/hot cot, phototherapy lamp, appropriate sized Ambu bag, oxygen supply/concentrator, appropriate sized face mask available, CPAP system, multi-function monitor, pulse oximeter, nasogastric tubes, glucometer, suction apparatus, thermometer, digital weighing scale |
| Routine neonatal care | Neonatal resuscitation, newborn assessment and immediate care, examination/screening/prevention/treatment of vertically transmitted infectious diseases in the newborn, early and exclusive breastfeeding, monitoring of the newborn before discharge, information and counselling for mothers |
| Case management of the sick newborn | Management of pre-term babies and low birth weight babies, neonatal sepsis, recognition and treatment of jaundice, management of convulsions in neonates, feeding needs for sick neonates |
| Monitoring and follow-up of sick newborns | Monitoring of individual progress, monitoring by nurses, reassessment by physicians and follow-up |

***Study sites and administering the facility assessment***

The facility surveys were conducted health facilities providing secondary-level care in three districts in southern Malawi in November 2017. These three districts represent a variety of health management structures available in Malawi (see table 3). Mission hospitals provide between 30-40% of the healthcare in Malawi and are private, not-for-profit facilities under the Christian Health Association of Malawi (CHAM). Maternal and child health services are under a service agreement with the Malawian government and provided free to patients at the mission hospitals. The four health facilities represent a spectrum of district-level facilities for strengthening newborn care capacities and the roll out of innovative neonatal technologies.

***Table 3: Study site characteristics***

|  | **District 1 –**  **Government hospital** | **District 2 –**  **Mission hospital** | **District 2 –**  **Primary health centre** | **District 3 –**  **Government hospital** |
| --- | --- | --- | --- | --- |
| Health management structure | Government district hospital serving as the regional referral centre delivering secondary-level care | Missing hospital serving as the regional referral centre delivering secondary-level care | Primary health centre referring to the mission hospital | Government district hospital serving as the regional referral centre delivering secondary-level care |
| Geographic zones | Southwest Malawi | Southeast Malawi | Southeast Malawi | Southwest Malawi |

The four steps in conducting the hospital assessment as outlined by the WHO Integrated Maternal, Neonatal and Child Quality of Care Assessment and Improvement Tool (version April 2014) are:

1. An introductory meeting with hospital administrators and staff
2. A walk-through in the health facility to obtain a sense of the hospital organization and areas to revisit for the assessment
3. Conducting the assessment
4. Assessors meeting where data collectors meet and debrief about key observations and findings.

The assessment involved observations of practices and availability of infrastructure, equipment and supplies as well as interviewing relevant health professionals, such as the nurse-in-charge of the ward and laboratory technicians, following the structured checklists in the tool.

Using a series of structured checklists, each aspect of care was observed and scored from one to five.

- 5 = good practice complying with standards of care
- 4 = little need for improvement to reach standard of care
- 3 = some need for improvement to reach standards of care
- 2 = considerable need for improvement to reach standards of care
- 1 = services not provided, totally inadequate care or potentially life-threatening practices

Scores are added up at the end of each section and an average calculated for the section. Assessors have space at the end of each section to provide some comments on the context of the score and any key issues within the topic area. There is also space to provide comments on the main strengths and weaknesses observed for the section.

Essential medicines are evaluated on a score of one to three from one being not available, two being available to not reliably stocked and may be expired or close to expiration, and three being readily available and not expired.

Each facility assessment was manually entered by two independent people into a RedCAP database (Vanderbilt University, Nashville, United States) and results compared to reduce inaccuracies in data entry and interpretation. Descriptive statistics on Excel (Microsoft, Redmond, United States) were calculated to summarize average scores for each area of care.

**Qualitative dataset**

***Bubble continuous positive airway pressure (bubble CPAP)***

Neonates with respiratory distress can be effectively managed by continuous positive airway pressure (CPAP). CPAP supports the newborn’s breathing effort by delivering a continuous regulated positive air pressure throughout the breathing cycle, therefore protecting the airways from collapse after expiration. Bubble CPAP (Bcpap) is a simple and relatively inexpensive form of CPAP. In Bcpap, positive pressure is generated by a continuous airflow from a source, such as an air compressor. The air pressure is regulated through submerging an end of the expiration tubing into water and the depth of the tube in the water determines the pressure in the system (18,20). This improves the functional residual lung capacity, the volume of air left in the lungs to the continuous positive airway pressure and improves the surface area available for gas exchange, thereby increasing oxygenation and reducing the work of breathing for the neonate.(18,20). Bubble CPAP therefore, represents a safe and cost-effective method which contributes to the reduction of neonatal mortality rates in low- and middle-income countries (13–15).

There are a number of different bubble CPAP systems. These range from improvised disposable water bottles, low cost standalone versions designed for resource-constrained health settings, to conventional commercial standalone systems. The Pumani bubble CPAP is a low-cost standalone version initially tested at Queen Elizabeth Central Hospital (QECH) in Malawi in 2012 (16) and then scaled up to all tertiary and secondary-level hospitals in Malawi from 2012 to 2017.

***Developing the interview guide***

A scoping literature review and preliminary stakeholder consultations were conducted with nurses, physicians and administrators at the hospitals as well as staff from Rice Nest 360 (Rice 360 Institute for Global Health) who developed the Pumani bubble CPAP system and supported its scale-up in Malawi. Based on the review and stakeholder consultations, a semi-structured interview guide was developed covering aspects of health worker training, process of initiating a neonate on bubble CPAP, monitoring a neonate on bubble CPAP, health worker perceptions of the system and experiences with caregivers. The interview guide was piloted with several nurses and physicians at a tertiary-level hospital who had experience with bubble CPAP. Pilot interviews were used to refine phrases of questions, support training data collectors on key areas to probe on and was not included in the final dataset. The interview guide was translated into Chichewa, the predominant local language in Malawi, prior to data collection.

***Data collection and analysis***

The 30 to 60 minute long semi-structured interviews were conducted in June to August 2018 at a tertiary hospital and three secondary-level hospitals in southern Malawi. The three secondary-level hospitals are the same as those covered in the facility assessment and coded with the same label for cross-referencing. The interviews covered training, initiation, monitoring, differences in opinions, perception and personal experiences, and perception on parental understanding of bubble CPAP. Face-to-face interviews were conducted by trained Malawian researchers at the health facilities in a private setting with 46 health workers purposefully sampled to obtain a wide range of perspectives. These included nurses, clinical officers, district health management (district health officer, district medical officer and district nursing officer), pediatric consultants and registrars. Nurses were interviewed at both the district hospitals (secondary-level health facility) and central hospital (tertiary-level health facility). Clinical officers and district health management were only interviewed at the district hospital as clinicians and management at the secondary-level facility while pediatric consultants and registrars are only available at the central hospital. Interviews were conducted in English, the language of instruction for medical professions in Malawi, and Chichewa, the local language in Malawi according to the participant’s preference.

Interviews were audio recorded, transcribed in verbatim and Chichewa phrases translated into English. Completed transcripts were sent to the transcription coordinator for review with audio to ensure quality and transcripts were uploaded to Nvivo 12 (QSR International, Melbourne, Australia) as a data management program for qualitative coding. Two qualitative researchers developed a codebook after familiarizing themselves with the transcripts and in discussion with data collectors. A third qualitative researcher completed the coding, which was independently reviewed by the first two qualitative researchers for quality and completeness.

**Technical validation**

The validity of the facility survey is supported by supervision of the data collection process and review of the data by neonatal health experts in Malawi and Canada (DMG, KW). Data collectors were debriefed regarding the process and the data obtained was entered into the database in duplicate by two independent staff and cross-checked for validity. Additionally, similar results as the previous delivery of the WHO Integrated Maternal, Neonatal and Child Quality of Care Assessment and Improvement Tool (version April 2014) conducted in five districts in Malawi, including two districts in southern Malawi (4), supports a validity of process. Furthermore, results were discussed with district health management and service providers involved in newborn care in each of the study sites who confirmed identified gaps during preliminary discussions around solutions.

The validity of the qualitative dataset is supported by the supervision of the data collection process, review of the data and supervision of data analysis by qualitative research experts in Malawi and Canada (MWK, ALNM). A native speaker of Chichewa who is fluent in English checked transcripts for quality against audio recordings. Qualitative results were presented at pediatric rounds at Queen Elizabeth Central Hospital with clinicians who confirmed themes.

**References**

1. Kawaza K, Kinshella MLW, Hiwa T, Njirammadzi J, Banda M, Vidler M, et al. Assessing quality of newborn care at district facilities in Malawi. BMC Health Serv Res. 2020 Mar 18;20(1):227.

2. Nyondo-Mipando AL, Kinshella MLW, Bohne C, Suwedi-Kapesa LC, Salimu S, Banda M, et al. Barriers and enablers of implementing bubble Continuous Positive Airway Pressure (CPAP): Perspectives of health professionals in Malawi. Ameh CA, editor. PLoS One. 2020 Feb 13;15(2):e0228915.

3. Salimu S, Kinshella MLW, Vidler M, Banda M, Newberry L, Dube Q, et al. Health workers’ views on factors affecting caregiver engagement with bubble CPAP. BMC Pediatr. 2020 Apr 23;20(1):180.

4. Smith H, Asfaw AG, Aung KM, Chikoti L, Mgawadere F, d’Aquino L, et al. Implementing the WHO integrated tool to assess quality of care for mothers, newborns and children: results and lessons learnt from five districts in Malawi. BMC Pregnancy Childbirth. 2017 Aug 25;17(1):271.

5. World Health Organization. Department of Child and Adolescent Health and Development. Health Facility Survey: tool to evaluate the quality of care delivered to sick children attending outpatient facilities. Vol. 1, World Health. 2003.

6. Health Organization W, Office for Europe R. Hospital Care for Mothers and Newborns: Quality Assessment and Improvement Hospital care for mothers and newborn babies: quality assessment and improvement tool [Internet]. 2014 [cited 2020 Jan 3]. Available from: http://www.euro.who.int/pubrequest

7. Organization WH. Integrated Maternal, Neonatal and Child Quality of Care Assessment and Improvement Tool: Draft. 2014.

8. World Health Organization. Regional Office for South-East Asia. Assessment tool for hospital care: improving the quality of care for reproductive, maternal, neonatal, child and adolescent health in South-East Asia. 2016.

9. UNFPA Sierra Leone. Maternal and Neonatal Quality of Care Baseline Assessment Report - Regent Community Health Centre [Internet]. 2018 [cited 2020 Jan 3]. Available from: https://sierraleone.unfpa.org/en/publications/maternal-and-neonatal-quality-care-baseline-assessment-report-regent-community-health

10. UNFPA Sierra Leone. Maternal and Neonatal Quality of Care Baseline Assessment Report - Jembe Community Health Centre [Internet]. 2018 [cited 2020 Jan 3]. Available from: https://sierraleone.unfpa.org/en/publications/maternal-and-neonatal-quality-care-baseline-assessment-report-jembe-community-health

11. UNFPA Sierra Leone. Maternal and Neonatal Quality of Care Baseline Assessment Report - Bo Government Hospital [Internet]. 2018 [cited 2020 Jan 3]. Available from: https://sierraleone.unfpa.org/en/publications/maternal-and-neonatal-quality-care-baseline-assessment-report-bo-government-hospital

12. UNFPA Sierra Leone. Maternal and Neonatal Quality of Care Baseline Assessment Report - Koribondo Community Health Centre [Internet]. 2018 [cited 2020 Jan 3]. Available from: https://sierraleone.unfpa.org/en/publications/maternal-and-neonatal-quality-care-baseline-assessment-report-koribondo-community

13. Ekhaguere OA, Mairami AB, Kirpalani H. Risk and benefits of Bubble Continuous Positive Airway Pressure for neonatal and childhood respiratory diseases in Low- and Middle-Income countries. Vol. 29, Paediatric Respiratory Reviews. 2019. p. 31–6.

14. Martin S, Duke T, Davis P. Efficacy and safety of bubble CPAP in neonatal care in low and middle income countries: a systematic review. Arch Dis Child Fetal Neonatal Ed. 2014 Nov 1;99(6):F495-504.

15. Carns J, Kawaza K, Liaghati-Mobarhan S, Asibon A, Quinn MK, Chalira A, et al. Neonatal CPAP for Respiratory Distress Across Malawi and Mortality. Pediatrics. 2019 Oct;144(4):e20190668.

16. Kawaza K, Machen HE, Brown J, Mwanza Z, Iniguez S, Gest A, et al. Efficacy of a low-cost bubble CPAP system in treatment of respiratory distress in a neonatal ward in Malawi. PLoS One Jan Malawi Med J. 2014;299(283):e86327.
